# Selected Essential Oils Act as Repellents Against the House Cricket, *Acheta domesticus*

**DOI:** 10.3390/insects17010106

**Published:** 2026-01-16

**Authors:** Torben K. Heinbockel, Rasha O. Alzyoud, Shazia Raheel, Vonnie D. C. Shields

**Affiliations:** Biological Sciences Department, Towson University, Towson, MD 21252, USA; torbenheinbockel@towson.edu (T.K.H.);

**Keywords:** olfaction, repellent, behavior, locomotion, essential oils, cricket, Orthoptera, antenna, green repellent, pest

## Abstract

The house cricket, *Acheta domesticus*, is found globally. It is an agricultural pest causing economic damage to a wide variety of crops including cereal seedlings, vegetable crops, fruit plants, and stored grains. Additionally, crickets act as mechanical vectors of pathogens by harboring bacteria, fungi, viruses, and toxins, causing foodborne illnesses. They can contaminate stored grains, packaged foods, or animal feed due to deposition of their feces, lowering the quality of the food and creating food safety risks. Synthetic insect repellents, such as pyrethroids and carbamates, have been used previously in integrated pest management practices to control crickets. Though successful as repellents, they have been associated with health and environmental risks and concerns. The use of organic green repellents, such as plant essential oils, may be a viable alternative in pest management practices. In this study, we tested the effects of 27 plant-based essential oils on the behavior of *A. domesticus*. *A. domesticus* were introduced into an open arena to allow them unrestricted movement. A transparent plastic bottle containing an essential oil treatment was placed in the arena to allow voluntary entry by the crickets. Following a predetermined observation period, the number of crickets that entered the bottle was recorded, and percent entry was calculated as the proportion of individuals inside the bottle relative to the total number in the arena. Analysis of the percentage entry into the bottles allowed for a comparative assessment of repellency of the selected essential oils examined in this study. Essential oils that elicited high levels of entry into the bottle were categorized as having weak or no repellency, while those that demonstrated reduced entry were classified as moderate or strong repellents. Our results indicated that *A. domesticus* responded with strong repellent behavior to nearly half of the essential oils tested, while four essential oils and two synthetic repellents evoked no significant repellent responses. Four strong repellent essential oils, namely peppermint, rosemary, cinnamon, and lemongrass, were tested at different concentrations and showed a clear dose-dependent repellent effect. The results suggest that selected essential oils can be useful in the development of more natural “green” insect repellents.

## Simple Summary

The aim of this study was to determine the behavioral responses of house crickets, *Acheta domesticus*, to various essential oils. We tested a panel of 27 essential oils and hypothesized that some would act as strong repellents, while others would have weak or no significant effects. In addition to the essential oils tested, we examined two synthetic repellents which elicited no significant repellent effects. This study is important in contributing to our current understanding of insect repellency and the use of naturally derived plant compounds, in lieu of their synthetic counterparts, to serve as repellents against insect pests.

## 1. Introduction

While crickets can be considered a promising novel food source in several regions of the world, including Africa, South America, Asia, and Oceania [1], they are also agricultural pests, disease vectors, and noise nuisances in household settings [2]. While they are not typically associated with the transmission of diseases, as other arthropods such as ticks, mosquitoes, and cockroaches, they still raise significant concern with respect to food contamination and hygiene (i.e., stored grains, flour, etc.), crop, fabric and paper damage, and annoyance attributed to their nocturnal chirping [3]. In addition, cricket home infestations are often indicators of poor sanitation and/or structural inadequacies to home dwellings. More specifically, they can enter garbage cans to feed on trash due to their omnivorous feeding preference, thereby potentially contaminating food sources with their fecal matter. Therefore, finding safe pest management practices, e.g., [4,5,6], to control house crickets and other arthropods that carry diseases is critical. While the application of baits and sprays containing synthetic chemicals, such as pyrethroids, carbamates, neonicotinoids, isooxazolines, and organophosphates, are options, these applications can pose problems and result in drawbacks such as toxicity to adults, children, and pets, potential build-up of harmful chemical residues in the environment, and increased chance of resistance to insect populations, e.g., [7,8,9,10]. In addition, commonly used household methods to dispose of crickets have proven to be relatively ineffective. These include glue boards, baited cornmeal, diatomaceous earth, vacuum cleaner hose suction, sprays, and baits laced with synthetic chemicals [11]. Considering this, there is a chemical and ecological need to find plant-derived alternatives, i.e., natural, “green” repellents, as candidates in integrated pest management.

*A. domesticus*, like other insects, use their chemical senses (smell and taste) for orientation, food selection, and mate finding. To detect olfactory environmental cues, they possess a pair of antennae bearing antennal sensory organs or sensilla [12]. These sensilla contain receptor neurons which encode and process olfactory stimuli. Such stimuli can trigger behaviors, such as orientation toward food and mating partners or avoidance of predators [13]. In this study, the objective was to test a panel of essential oils on house cricket behavior.

Essential oils are lipophilic, volatile, organic, and “green” (i.e., environmentally friendly) compounds representing mixtures of volatile secondary metabolites synthesized by aromatic plants [14]. Plants use these compounds to protect themselves against predators, such as insects and pathogens, and they can also serve as signaling compounds. These compounds include terpenes, terpenoids, aldehydes, phenols, etc., and can be extracted from plants using several methods (i.e., steam distillation, cold pressing, solvent extraction) [15]. In traditional medicine, they have been used as fragrances, antiseptics, and remedies. Essential oils comprise hydrocarbon molecules and can be classified as terpenes, alcohols, esters, aldehydes, ketones, phenols, oxygenated compounds, monoterpene alcohols, sesquiterpene alcohols, aldehydes, esters, lactones, coumarins, ethers, and oxides [14]. It is noted, however, that the most active compounds fall into two chemical groups: terpenoids (monoterpenoids and sesquiterpenoids) and phenylpropanoids [14,16].

Volatile molecules emanating from essential oils can be detected by olfactory receptor cells housed within olfactory sensory organs (or sensilla) in insect antennae [13]. Such molecules may be responsible for regulating insect behavior. In the case of essential oils, recent evidence has demonstrated that they can serve as olfactory repellents [17,18]. Essential oils can disrupt insects’ olfactory-guided locomotory behavior, making it difficult for them to detect humans. In addition, they offer antimicrobial activity as they contain active compounds like terpenes, phenols, and aldehydes, which inhibit a broad range of microorganisms, including bacteria, fungi, and viruses [19,20,21,22].

Insect repellents differ from insecticides in their mode of action as they do not kill insects, but they deter them approaching, landing, or establishing residence [23]. This has important relevance to cricket management, as the primary goal is to protect household materials and prevent food contamination rather than eradicate insect populations. Due to the volatile nature of repellents, their odors do not persist on a long-term basis in the environment, thereby requiring frequent reapplication [24]. Additionally, there is need to explore novel methods to advance their formulation to ensure stabilization and prolonged action. In practice, this may require techniques, such as microencapsulation, nano emulsification, and the use of polymer-based slow-release methods to optimize their efficacy [25] as well as the development of more portable applications (e.g., sprays) tailored to cricket control.

With growing interest in plant-based essential oils as environmentally friendly insect repellents, there is growing knowledge and evidence evaluating their behavioral repellency against arthropods. Comparative assessments of multiple essential oils and their dose-dependent effects on house cricket behavior remain largely unexplored. To address this gap, the present study systematically evaluated the behavioral responses of *A. domesticus* to a diverse panel of 27 plant-based essential oils, which allowed unrestricted movement and voluntary exposure. We hypothesized that some of the essential oils tested would act as strong repellents, while others would have weak or no significant effects. Additionally, two well-known synthetic repellents were also tested and elicited no significant repellent effect. By providing a comparative ranking of essential oils based on behavioral repellency and demonstrating dose-dependent effects for select compounds, this study contributes novel evidence supporting the potential use of essential oils as effective green alternatives to synthetic insect repellents in cricket management.

Eco-friendly green repellents are needed as safer, more effective, and sustainable pest control solutions compared to synthetic chemical alternatives, which pose risks to human and animal health, food safety, and the environment.

## 2. Materials and Methods

### 2.1. Insect Rearing and Maintenance

Adult *A. domesticus* (Timberline, Marion, IL, USA) were housed in a controlled environment maintained at 22 °C ± 2 °C with a 16:8 light–dark cycle. They were provided ad libitum access to cricket food and Easy Water (Timberline, Marion, IL, USA) prior to experimentation. Female and male crickets were randomly selected. Each cricket was naïve to the specific odorant prior to testing and was discarded following its use to prevent any potential bias from prior odorant exposure.

### 2.2. Repellency Bioassay

**Figure 1:**
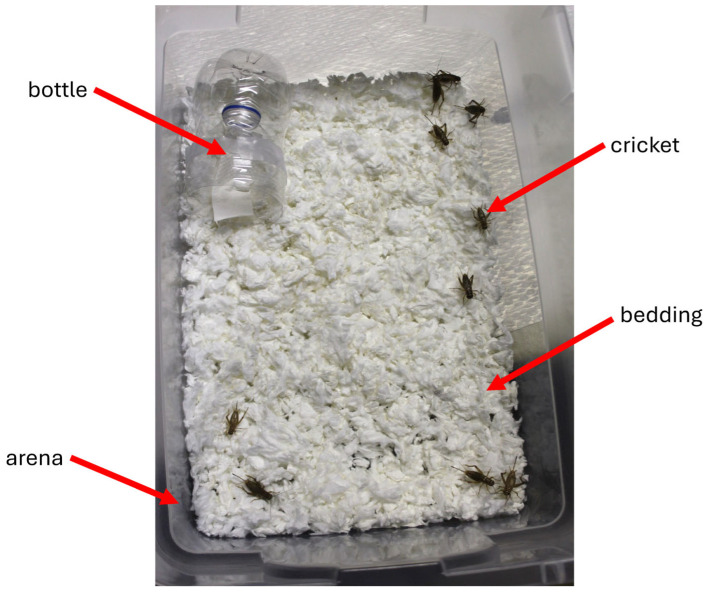
Top-down view of the behavioral bioassay showing the inverted bottle, crickets, bedding, and plastic arena (lid removed). Ten crickets were allowed to explore freely in the arena during the experiment. The inverted bottle contained either an essential oil (test) or alcohol (control).

Testing occurred in large plastic containers (Figure 1). The open arena (i.e., container) measured 42.9 cm × 29.2 cm × 14.9 cm (Sterilite, Townsend, MA, USA) and was filled with 350 g of unscented paper bedding material (Kaytee Products, Inc., Chilton, WI, USA). Plastic water bottles (500 mL) were cut in half, and the top half was inverted into the bottom half and secured with clear tape. This design prevented the crickets from escaping during testing. Filter paper disks (2.4 cm diameter circles) (Whatman/Cytiva, Marlborough, MA, USA) served as carriers for the selected essential oils (E.O.s) (test substance) which were dissolved with pure ethyl alcohol (200 proof; Pharmco Products Inc., Brookfield, CT, USA). For control experiments, only pure ethyl alcohol was applied to the filter paper. Experimental bioassays for control and test groups were conducted in separate locations to ensure no cross-contamination of the odorant-containing bottles and control bottles. To determine dose–response dynamics, we tested four different concentrations (0.05 µg, 0.5 µg, 5 µg, and 50 µg). Doses were selected based on preliminary data.

For each experiment, 10 crickets were introduced into each arena which maintained consistency of sample size. Crickets were allowed to acclimate for four minutes prior to testing. For each essential oil tested, the experiment was replicated at least 10 times with 10 crickets per container (N ≥ 100 crickets).

### 2.3. Statistical Analyses

Orientation towards or away from the odorant source was assessed to determine the effects of the odorant on cricket behavior and to calculate the percentage of crickets that entered the bottle or remained in the open arena of the container. The degree of repellency of a particular essential oil tested was expressed as the percentage of crickets that had entered a bottle (i.e., percentage entry) during the trial period. The percentage entered was calculated by observing the total number of crickets that had entered test bottles and then dividing this number by the overall number of crickets tested during a particular trial. This number was then multiplied by 100. Bottles containing the control compound were treated in a similar manner to those containing test compounds.

$$\%\;\mathrm{entered}=\left(\frac{N\;\mathrm{entered}}{N}\right)\times 100$$

N _entered_ = Number of crickets that entered bottles containing test or control compounds

N = Total number of crickets tested during trial.

All bioassays were run in the evening for 6 h, as crickets are most active in the evenings due to their nocturnal lifestyle and to ensure a sufficient observation period for behavioral responses. Hourly observations were also made. Upon completion of each experiment, all crickets and bottles used for experiments were disposed of to prevent potential cross-contamination.

An evaluation of the strength of repellency for all the essential oils tested was carried out by assigning three ranges of percent entry: 0–25% (strong repellent), >25–50% entry (medium repellent), and >50%–<70% entry (weak repellent). Statistical analysis of data was performed using a table of cross-categorized frequency data for a chi-square test of association (VassarStats Website for Statistical Computation, http://vassarstats.net/) (accessed 24 December 2025). Yates values were reported and corrected for continuity, and probability estimates were non-directional. Statistical significance was considered for *p*-values smaller than 0.05.

### 2.4. Chemicals and Reagents

Essential oils (100% pure and organic) were purchased from Cliganic, Walnut, CA, USA (peppermint, *Mentha piperita*, rosemary, *Rosmarinus officinalis*, tea tree, *Melaleuca alternifolia*, lemon grass, *Cymbopogon citratus*, lavender, *Lavandula angustifolia*, frankincense, *Boswellia carterii*, orange, *Citrus sinensis*), Anjou, Fremont, CA, USA (sage, *Salvia officinalis*, cinnamon, *Cinnamomum verum*, bergamot, *Citrus bergamia*, lemon, *Citrus limon*, grapefruit, *Citrus paradisi*, patchouli, *Pogostemon cablin*, geranium, *Pelargonium graveolens*, cypress, *Cupressus sempervirens*, ylang ylang, *Cananga odorata*, palmarosa, *Cymbopogon martinii*), Aromappeal, Holbrook, NY, USA (basil, *Ocimum basilicum*, citronella, *Cymbopogon nardus*, lemon eucalyptus, *Corymbia citriodora*, clove, *Syzygium aromaticum,* eucalyptus, *Eucalyptus globulus*), Now, Bloomingdale, IL, USA (wintergreen, *Gaultheria procumbens*), Plant Therapy, Twin Falls, ID, USA (juniper berry, *Juniperus communis*), Aura Cacia, Urbana, IA, USA (sweet fennel, *Foeniculum vulgare*), Edens Garden, San Clements, CA, USA (catnip, *Nepeta cataria*), and Sedbuwza, Hagerstown, MD, USA (coffee, *Coffea arabica*) (Table 1). Each essential oil was pipetted onto a filter paper and placed at the bottom of each bottle. Four minutes were allowed to elapse prior to placing each bottle in an arena. The bottle (containing the test odorant or the control) was positioned at random locations in the containers to prevent bias. To compare essential oil repellency with repellent effects of known synthetic repellents, IR3535 and DEET were tested with the same bioassay design.

## 3. Results

### 3.1. Essential Oils as Repellent Compounds

In this study, we tested the effects of 27 essential oils (i.e., ylang ylang, sweet fennel, frankincense, juniper berry, cypress, wintergreen, geranium, cinnamon, sage, peppermint, basil, rosemary, lavender, catnip, patchouli, tea tree, lemon eucalyptus, clove, eucalyptus, citronella, lemongrass, palmarosa, coffee, bergamot, lemon, grapefruit, orange) on the behavior of *A. domesticus*. These oils belonged to 12 different plant families (i.e., Annonaceae, Apiaceae, Burseraceae, Cupressaceae, Ericaceae, Geraniaceae, Lauraceae, Lamiaceae, Myrtaceae, Poaceae, Rubiaceae, and Rutaceae) (Table 1) and fell into four main chemical groups: monoterpenes/monoterpenoids, diterpenes/diterpenoids, sesquiterpenes/sesquiterpenoids, and aromatics (phenylpropanoids, phenolic acids, benzenoids) (Table 2).

**Figure 2:**
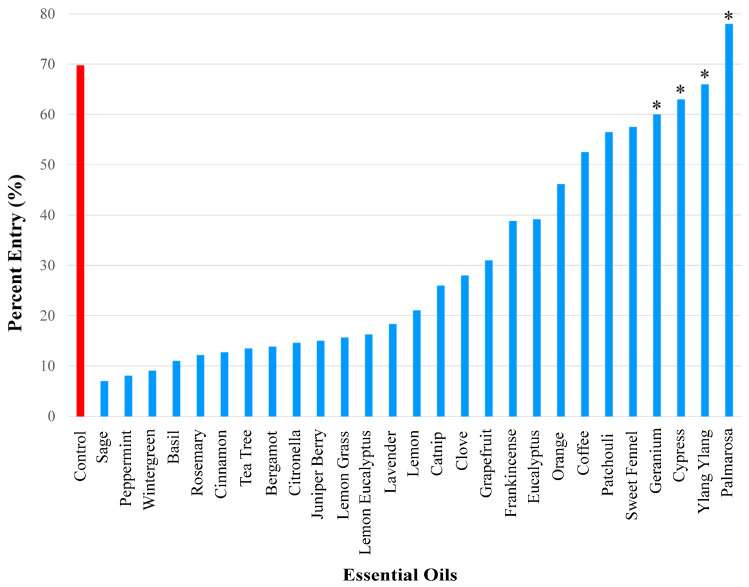
Overall results showing the degree of repellency elicited by 27 essential oils. Repellency was expressed as percent entry into a plastic bottle containing a test essential oil. Ethanol was used for all control experiments. Percent entry for test compounds was compared with that for the control compound (69.8%). Asterisks indicate essential oils that did not elicit significant repellency.

**Figure 3:**
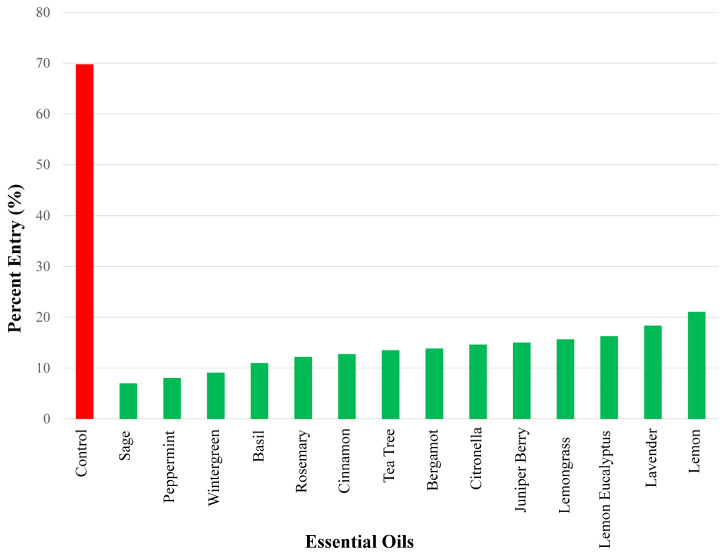
Repellency results of the essential oils exhibiting the strongest repellency (i.e., percent entry range: 0–25%) using a 50 µg dose are shown. Repellency was expressed as percent entry into a plastic bottle containing the test compound (i.e., essential oil). Ethanol was used for all control experiments. Percent entry for test compounds was compared with that for the control compound (69.8%). All essential oils in this figure elicited significant repellency.

**Figure 4:**
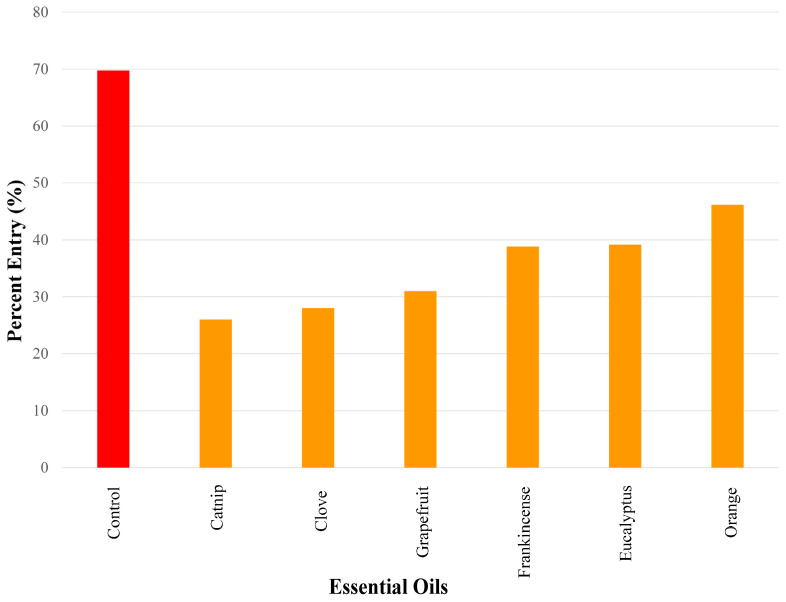
Results of repellency tests showing the essential oils that exhibited medium repellency (i.e., percent entry range > 25–50%) using a 50 µg dose. Repellency was expressed as percent entry into a plastic bottle containing the test compound (i.e., essential oil). Ethanol was used for all control experiments. Percent entry for test compounds was compared with that for the control compound (69.8%). All essential oils in this figure elicited significant repellency.

**Figure 5:**
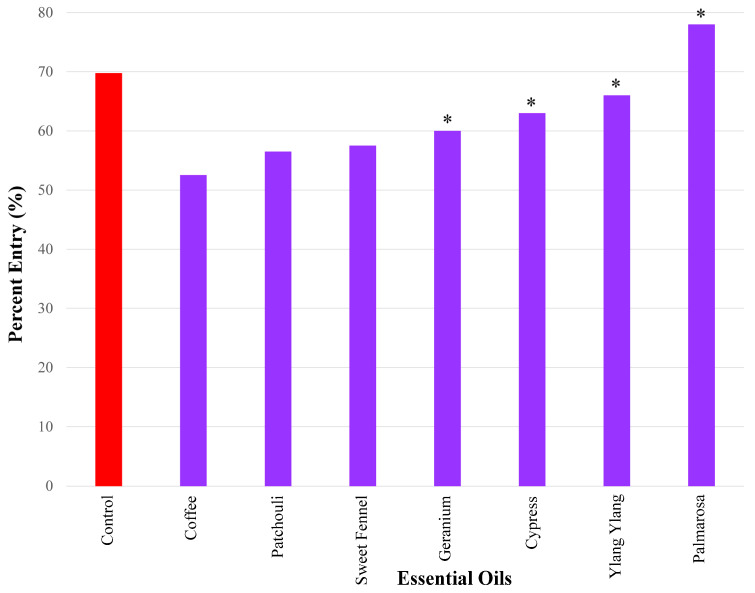
Results of repellency tests, showing the essential oils that exhibited weak repellency (i.e., percent entry range (>50%–<70%) using a 50 µg dose. Only coffee, patchouli, and sweet fennel elicited significant repellency. Four compounds did not elicit significant repellency, indicated by the asterisks, namely geranium, cypress, ylang ylang, and palmarosa. Repellency was expressed as percent entry into a plastic bottle containing the test compound (i.e., essential oil). Ethanol was used for all control experiments. Percent entry for test compounds was compared with that for the control compound (69.8%).

These 27 essential oils served as possible repellents against *A. domesticus* (Figure 1). Test data was compared with control results. During control experiments, when no essential oil was present on the filter paper, 69.8% of the crickets in the container entered the inverted bottle. This served as the baseline to assess repellency of the tested essential oils. The strength of repellency was categorized by assigning essential oils to one of three ranges of percent entry: 0–25% entry (strong repellent), >25–50% entry (medium repellent), and >50%–<70% entry (weak repellent) (Figure 2, Figure 3, Figure 4 and Figure 5). Four of the twenty-seven essential oils tested, namely geranium, cypress, ylang ylang, and palmarosa, elicited no repellent effect. Yates values were reported and corrected for continuity, and probability estimates were non-directional. Statistical significance was considered for *p*-values smaller than 0.05 and Yates values were reported for the following essential oils: sage (Yates, 26.79, *p* < 0.0001), peppermint (Yates, 210.87, *p* < 0.0001), wintergreen (Yates, 103.06, *p* < 0.0001), basil (Yates, 90.49, *p* < 0.0001), rosemary (Yates, 148.24, *p* < 0.0001), cinnamon (Yates, 141.16, *p* < 0.0001), tea tree (Yates, 129.24, *p* < 0.0001), bergamot (Yates, 97.39, *p* < 0.0001), citronella (Yates, 94.66, *p* < 0.0001), juniper berry (Yates, 78.53, *p* < 0.0001), lemon grass (Yates, 128.79, *p* < 0.0001), lemon eucalyptus (Yates, 128.75, *p* < 0.0001), lavender (Yates, 78.04, *p* < 0.0001), lemon (Yates, 92.12, *p* < 0.0001), catnip (Yates, 99.71, *p* < 0.0001), clove (Yates, 46.8, *p* < 0.0001), grapefruit (Yates, 39.64, *p* < 0.0001), frankincense (Yates, 34.8, *p* < 0.0001), eucalyptus (Yates, 27.92, *p* < 0.0001), orange (Yates, 17.58, *p* < 0.0001), coffee (Yates, 13.06, *p* < 0.0003), patchouli (Yates, 7.09, *p* < 0.0077), sweet fennel (Yates, 6.06, *p* < 0.0138), geranium (Yates, 3.71, *p* < 0.05408), cypress (Yates, 1.11, *p* < 0.2921), ylang ylang (Yates, 0.28, *p* < 0.5967), and palmarosa (Yates, 0.01, *p* < 0.9203). All the essential oils in Figure 2 were statistically significant from the control and elicited repellency, except for geranium, cypress, ylang ylang, and palmarosa. Percent entry values are discussed for each group of essential oils (i.e., strong, medium, weak repellency), in Section 2.2, Section 2.3 and Section 2.4. Importantly, when known synthetic repellents, such as IR3535 and DEET, were tested, they elicited no significant repellency (percent entry, 59%, Yates, 3.01, *p* < 0.0827 and percent entry, 68%, Yates, 0.03, *p* < 0.8625), respectively.

### 3.2. Essential Oils Eliciting Strong Repellent Responses

In terms of percent entry into bottles with test compounds, we found 14 essential oils that elicited between 7 and 21.1% entry. In ascending order, the strongest repellency using a 50 µg dose was found for the following essential oils (Figure 3): sage (7.00%), peppermint (8.06%), wintergreen (9.09%), basil (11.0%), rosemary (12.2%), cinnamon (12.7%), tea tree (13.5%), bergamot (13.8%), citronella (14.6%), juniper berry (15.0%), lemon grass (15.%), lemon eucalyptus (16.3%), lavender (18.3%), and lemon (21.1%).

### 3.3. Essential Oils Eliciting Medium Repellent Responses

The essential oils that elicited medium repellency using a 50 µg dose in ascending order were as follows: catnip (26.0%), clove (26.0%), grapefruit (31.0%), frankincense (38.8%), eucalyptus (39.2%), and orange oil (46.2.%) (Figure 4).

### 3.4. Essential Oils Eliciting Weak Repellent Responses

The essential oils that caused the weakest repellency using a 50 µg dose in ascending order were as follows: coffee (52.5%), patchouli 56.5%), and sweet fennel (57.5%). Geranium (60%), cypress (63%), ylang ylang (66%), and palmarosa (78%) did not elicit significant repellency (Figure 5).

### 3.5. Dose–Response Curves

**Figure 6:**
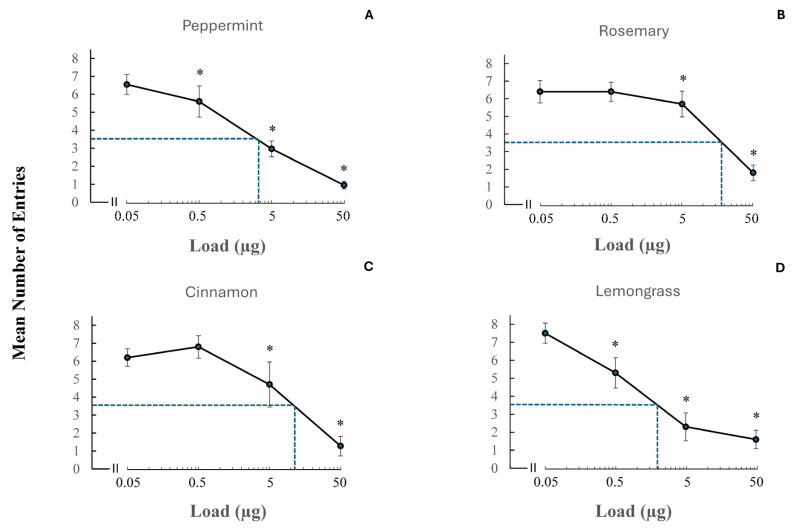
Dose–response curves for four essential oils eliciting strong repellency, namely (**A**) peppermint, (**B**) rosemary, (**C**) cinnamon, and (**D**) lemongrass at 0.05 µg, 0.5 µg, 5 µg, and 50 µg. Each test compound was applied to a filter paper disk which was then placed in an empty bottle. Control experiments were run in a similar manner by applying alcohol to a filter paper disk. The hatched line indicates the RD_50_ concentration (concentration which represented 50% of the insect population that was repelled). The RT (repellency threshold) concentrations represent the lowest concentrations at which repellency was significantly different from that of the control and indicated by the asterisks.

From those essential oils eliciting strong repellency, four were selected (i.e., peppermint, rosemary, cinnamon, and lemongrass) to determine their dose–response dynamics at four different concentrations (0.05 µg, 0.5 µg, 5 µg, and 50 µg) (Figure 6). In all cases, we observed a decrease in the mean number of entries into the test bottle with increasing concentration. Differently shaped sigmoid curves were noted. The RT (repellency threshold) concentration (i.e., lowest concentration at which repellency was significantly different from that of the control) was determined for each of the four essential oils. Statistical significance was considered for *p*-values smaller than 0.05 and Yates values were reported for these four essential oils and doses: (Yates, 150.65, *p* < 0.0001, Yates, 77,2, *p* < 0.0001, Yates, 5.02, *p* < 0.0251, Yates, 0.65, p < 0.4201 for 50 µg, 5 µg, 0.5 µg, and 0.05 µg peppermint, respectively, Yates, 70.18, *p* < 0.0001, Yates, 4.3, *p* < 0.0381, Yates, 0.77, *p* < 0.3802, Yates, 0.65, *p* < 0.4201 for 50 µg, 5 µg, 0.5 µg, and 0.05 µg rosemary, respectively, Yates, 59.39, *p* < 0.0001, Yates, 13.87, *p* < 0.0002, Yates, 0.03, *p* < 0.8625, Yates, 1.5, *p* < 0.2207 for 50 µg, 5 µg, 0.5 µg, and 0.05 µg cinnamon, respectively, and Yates, 48.74, *p* < 0.0001, Yates, 57.37, *p* < 0.0001, Yates, 7.5, *p* < 0.0062, Yates, 0.67, *p* < 0.4131 for 50 µg, 5 µg, 0.5 µg, and 0.05 µg lemongrass, respectively. In the case of both peppermint and lemongrass, the RT was 0.05 µg, whereas for rosemary and cinnamon, this value was 0.5 µg. We estimated the RD_50_ concentration (concentration at 50% of the population of insects in the test group were repelled). As the control compound elicited 69.8% (~70%) and not 100% entry into control bottles, the concentration of 35% (i.e., 50% of control entries) was used as the RD_50_ value for test compounds. Since, on average, seven out of ten insects entered the bottle in control experiments, the RD_50_ value was set to 3.5 number of entries (see y axes in Figure 6). In the case of lemon grass, peppermint, cinnamon, and rosemary, these RD_50_ values were found to be approximately 1.1 µg, 2.3 µg, 6.8 µg, and 10 µg, respectively.

The data presented in the four dose–response curves suggested three main findings: (a) peppermint and lemongrass were the most repellent compounds; (b) while lemongrass appeared to have a slightly lower RD_50_ concentration than peppermint (1.1 µg versus 2.3 µg, respectively), it was evident that more crickets were initially repelled by peppermint at the highest dose tested (i.e., 50 µg), and (c) rosemary was the least potent compound tested among the four potent repellents.

## 4. Discussion

Essential oils are plant-produced, bioactive secondary metabolites that are thought to be less toxic to beneficial insects and environmentally friendly alternatives to synthetic repellents. They are highly concentrated, volatile, fat-soluble, biodegradable, colorless mixtures of aromatic compounds used for defense and signaling purposes (e.g., deter pests and pathogens, attract pollinators, and communicate with other organisms) [14]. Essential oils have been found to demonstrate antimicrobial, antioxidant, and/or insect-repellent activity [19]. Some essential oils also interfere with insect odorant receptors (i.e., “olfactory interference”) and can disrupt navigation, oviposition, and feeding, leading to avoidance of food sources and a reduction in oviposition [20,24].

In this study, we determined the potential repellency of 27 essential oils using an olfactory test bioassay against *A. domesticus*. The effectiveness of repellency was determined by the number of crickets entering the plastic bottle containing the test compound. Repellency effectiveness was assigned to three groups, consisting of essential oils eliciting strong repellency (0–25% entry), medium repellency (>25–50% entry), and weak repellency (>50%–<70% entry).

Our results demonstrated that 14 of the 27 essential oils tested elicited strong repellency, namely sage, peppermint, wintergreen, basil, rosemary, cinnamon, tea tree, bergamot, citronella, juniper berry, lemongrass, lemon eucalyptus, lavender, and lemon. Medium repellent behavior was observed with catnip, clove, grapefruit, frankincense, eucalyptus, and orange. Weak repellent behavior was observed with coffee, patchouli, and sweet fennel. Geranium, cypress, ylang ylang, and palmarosa elicited no significant repellent effect. Similarly, synthetic repellents, IR3535 and DEET, elicited no significant repellency. Dose–response data from four essential oils that elicited strong repellent behavior in this study (i.e., peppermint, rosemary, cinnamon, and lemongrass) indicated that RT and RD_50_ values were similar for peppermint and lemongrass, as they were for rosemary and cinnamon.

The essential oils tested in this study comprised terpenes with non-oxygenated hydrocarbons built from isoprene units (C_5_H_8_). They were further differentiated as monoterpenes (C_10_ structures bearing two isoprene units; most common and highly volatile), sesquiterpenes (C_15_ structures bearing three isoprene units; less volatile), and diterpenes (C_20_ structures bearing four isoprene units; less common, less volatile, and more resin-like). Terpenoid families included monoterpenes, sesquiterpenes, and diterpenes (bearing a hydrocarbon skeleton and oxygen-containing functional groups), which included alcohols (-OH), aldehydes (-CHO), ketones (C=O), esters (-COO-), phenols (aromatic ring with an -OH group on the benzene ring), and ethers (R-O-R). Non-terpenoid families included phenylpropanoids (C_6_H_5_-CH_2_-CH_2_-R) and coumarins (benzopyrone compounds). Functional groups, like lactones (cyclic esters) and oxides (cyclic ethers) straddled both terpenoid and non-terpenoid classifications [27].

There is evidence in the literature that addresses some of the repellent properties of single compounds found with some essential oils tested in this study that elicited strong repellency. In the lemon-scented Australian gum tree, *Corymbia citriodora* (lemon eucalyptus), the monoterpene, p-menthane-3,8-diol (PMD), was found to offer long-lasting, deet-like efficacy/repellency in some mosquito species (e.g., *Aedes*, *Anopholes*, *Culex*, and *Ochlerotatus*) [29]. Indoor experiments with citronella essential oil diffusers, as well as derivatives, geraniol and linalool, demonstrated repellent effect against *Aedes aegypti* mosquitoes [30]. Lemongrass and citronella essential oils offered repellent protection from *Culex quinquefasciatus* mosquitoes [31]. Wu et al. [32] tested 60 essential oils, as well as eight of the most active constituents, when testing *Aedes albopictus* mosquitoes. These authors found cinnamon to be the most repellent (~77%), followed by lemongrass (~54%) and peppermint 2 (~42%). Citronella, bergamot, and sage were only ~29%, ~25%, and ~25%, respectively, as effective in comparison to cinnamon. These results differed to the results of the current study, as we found sage to be the strongest repellent followed by peppermint, rosemary, cinnamon, and citronella (see Figure 3). Interestingly Wu et al. [32] found that while peppermint 2 exhibited a potent repellent effect (~42%), its main constituents, namely p-menthone, menthol, and limonene, showed only moderate to low repellency, highlighting the importance of a synergistic effect among constituents which resulted in higher bioactivity as compared to isolated components [33].

The essential oils tested in this study that elicited the strongest repellency (i.e., 0–25% entry) were categorized into five main odor groups (Table 3). They were classified as monoterpenes and monoterpenoids (alcohols, esters, aldehydes, oxides, and ketones), phenylpropanoids (esters, aldehydes), and sesquiterpenes (Table 2). These essential oils fell into five main odor categories: camphor-like, minty, floral (sweet), citrus, spicy (woody), and pine-like (Table 3). Five (i.e., basil, rosemary, tea tree, sage, and lemon eucalyptus) of the fourteen essential oils that elicited the strongest repellency fell into the category of camphor-smelling, with two (i.e., peppermint and wintergreen) with a minty odor, two (lavender and bergamot) with a sweet floral odor, three (lemon, lemongrass, citronella) with a citrus/lemon odor, one (cinnamon) with (woody) odor, and one (juniper berry) with a pine-like odor (Table 3).

Some of the essential oils tested in this study (i.e., ylang ylang, sweet fennel, geranium, lemon eucalyptus, citronella, lemongrass, palmarosa, peppermint, basil, rosemary, lavender, catnip, patchouli, tea tree, clove, cinnamon, sage, juniper berry, cypress wintergreen, frankincense, coffee, and eucalyptus, lemon, orange, grapefruit) are known to exhibit both larvicidal and repellent activity in mosquitoes, blowflies, ticks, and store-product pest species. Studies on various insect and arthropod species have demonstrated that some essential oils serve as natural repellents including citronella and lemongrass (containing citronellal and geraniol, both mosquito repellents), catnip (nepetalactone), peppermint and clove (containing menthol and eugenol), and cinnamon and basil (containing eugenol and cinnamaldehyde) [34,35,36,37,38,39,40,41,42,43,44,45,46,47,48,49,50,51,52,53,54].

The effect of essential oils on target receptors, enzymes, and channels is key to understanding insecticidal activity and biological mechanisms of action. Examples include inhibiting gamma-aminobutyric acid (GABA), octopamine, and tyramine receptors, changes in acetylcholinesterase activity, interference with glutamate-gated chloride channels, disruption of growth, and effects on cuticular permeability. These effects result in dehydration or enhanced means for transdermal drug delivery. α-pinene carvacrol, limonene, menthol, menthone, and 1,8-cineole have been shown to interact with acetylcholinesterase activity, while eugenol and cinnamaldehyde have been shown to interact with octopamine and tyramine receptors. Carvacrol, pulegone, and thymol have been found to boost the inhibitory effect of GABA at its receptors without directly turning the receptor on by themselves. In addition, thymol and menthol were found to inhibit glutamate-gated chloride channels [55,56,57,58].

Essential oils exhibit measurable repellent activity across multiple insect species and assay types. While performance varies among oils and formulations, many show comparable short-term efficacy to synthetic repellents and often do so with lower ecological and toxicological risk. As resistance to conventional insecticides continues to rise and demand grows for eco-conscious solutions, essential oils represent a promising avenue for future repellent development. With further refinement of delivery systems and deeper investigation into active constituents, botanically derived repellents can become viable, sustainable alternatives to synthetic chemicals in integrated pest management strategies.

Few species, like *A. domesticus*, sit at a rare intersection where the same insect can be considered an indoor pest as well as a deliberately farmed protein source. On the one hand, when these insects enter homes, warehouses, and food facilities, they can become a nuisance through persistent chirping, creating unpleasant odorants during heavier infestations, and sanitation concerns from fecal deposits or dead insects. They can also contaminate stored foods and occasionally chew on paper, fabric, or packaging, which makes them unwelcome in any setting where cleanliness and product integrity matter. At the same time, *A. domesticus* are one of the most widely used edible insects and are increasingly valued as a protein source. Crickets grow quickly, reproduce efficiently, and can effectively convert feed into edible biomass, which is why they can be processed and incorporated into ingredients for animal feed, pet food, as well as human foods (e.g., cricket powder or flour to boost protein and micronutrients). In sustainability discussions, insect farming is also often highlighted because it requires less land and water than many conventional livestock systems. The link between these roles is that the traits that help crickets succeed as pests are the same traits that make them attractive for farming. That creates a shared challenge: production must be tightly managed. Containment, waste control, and biosecurity are essential to prevent escapes and to reduce odorant or disease risk. Food safety practices are especially important for human consumption. In short, *A. domesticus* are problematic when they are uninvited and unmanaged, but valuable when they are contained, hygienically produced, and used intentionally as food or feed. As cricket farming expands, so does the importance of biosecurity and containment at the interface between production and surrounding environments. Repellents could reduce escape-related nuisance and limit re-entry into facilities, supporting both pest prevention and responsible insect production. Current control strategies often emphasize exclusion and insecticides, but these can be incomplete or undesirable in certain environments. This motivates the search for deterrents that are effective, low-residue, and compatible with integrated pest management, positioning repellency as a key focus.

## 5. Conclusions

In this study, we determined the potential repellency of 27 essential oils using an olfactory test bioassay against *A. domesticus* and assigned them to three groups, namely those eliciting strong, medium, and weak repellency. Some essential oils elicited no significant repellent effect. When synthetic repellents, IR3535 and DEET, were tested, they were found to elicit no significant repellency. Essential oils eliciting the strongest repellency were organized into five main odor categories: camphor-like, minty, floral (sweet), citrus, spicy (woody), and pine-like. The effectiveness of these essential oils as repellents shows their potential as eco-friendly repellents against the house cricket, suggesting their suitability for integration into pest management methods.

A limitation of this study is the lack of GC-MS profiling of the tested materials. Without chemical characterization, we cannot confidently link repellency to specific volatile constituents, compare batches or chemotypes, or assess how natural variation in composition may have influenced performance. This may also limit reproducibility across laboratories and seasons, since the relative abundance of major and minor components may shift with source, storage conditions, and age. GC-MS analyses, ideally with internal standards and replicate lots, could help to quantify dominant compounds and connect behavioral outcomes to chemical drivers. A second limitation is the short experimental duration. While brief assays are well-suited for detecting immediate avoidance, they may not capture sustained effectiveness over time where repellency can decline as compounds evaporate or degrade. Extending assays across longer intervals with repeated measurements may help to detect decay in effect and possible habituation. It may lead researchers to be able to better quantify persistence under realistic conditions (hours to days) and allow a comparison of initial versus sustained repellency.

Finally, while lab assays reduce noise and improve internal validity, they may not translate directly into real-world environments where airflow, temperature, humidity, competing odors, refuge availability, and substrate differences can substantially alter insect movement and the concentration of airborne volatiles. The addition of field or semi-field trials may provide needed input to assess effectiveness under operational conditions and to define practical deployment parameters.

Essential oils can provide meaningful repellent effects. Future work should focus on improving real-world performance and interpretability. One priority could be in formulation development, since encapsulation and emulsification technology, as well as the development of gels, waxes, or controlled-release matrices, could stabilize volatile actives and extend repellency duration while reducing rapid dissipation. The safety evaluation should also accompany efficacy testing, including exposure and residue considerations, for indoor use, non-target impacts, irritation or sensitization risk, compatibility with food-handling environments, and existing IPM practices.

## Figures and Tables

**Table 1:** Essential oils tested in this study belonging to 12 plant families.

Plant Family	Essential Oils Included
Annonaceae	Ylang ylang
Apiaceae	Sweet fennel
Burseraceae	Frankincense
Cupressaceae	Juniper berry, Cypress
Ericaceae	Wintergreen
Geraniaceae	Geranium
Lauraceae	Cinnamon
Lamiaceae	Sage, Peppermint, Basil, Rosemary, Lavender, Catnip, Patchouli
Myrtaceae	Tea tree, Lemon eucalyptus, Clove, Eucalyptus
Poaceae	Citronella, Lemongrass, Palmarosa
Rubiaceae	Coffee
Rutaceae	Bergamot, Lemon, Grapefruit, Orange

Note: Resources used [26].

**Table 2:** Composition of essential oils tested.

Essential Oils	Monoterpenes/Oids	Diterpenes/Oids	Sesquiterpenes/Oids	Aromatics (Phenylpropanoids, Phenolic Acids, Benzenoids)	Major Constituents
Basil	√	-	minor	√	Monoterpenoid alcohols (linalool), phenylpropanoids (estragole, eugenol)
Bergamot	√	-	-	-	Monoterpenoid esters (linalyl acetate), monoterpenoid alcohols (linalool), coumarins (non volatile)
Catnip	√ (Iridoids)	-	-	-	Iridoids (monoterpenoid lactones, nepetalactone)
Cinnamon	-	-	-	√	Phenylpropanoid aldehydes (cinnamaldehyde)
Citronella	√	-	minor	-	Monoterpenoid aldehydes (citronellal), monoterpenoid alcohols (citronellol, geraniol), monoterpenoid esters
Clove	-	-	√	√	Phenylpropanoid phenols (eugenol), phenylpropanoid esters (eugenyl acetate), sesquiterpenes (*β*-caryophyllene)
Coffee	minor	√	-	√	Diterpenes (cafestol, kahweol), phenolic acid components as well
Cypress	√	-	√	-	Monoterpenes (*α*-pinene), sesquiterpenoid alcohols (cedrol)
Eucalyptus	√	-	-	-	Monoterpenoid oxides (1,8-cineole)
Frankincense	√	√	√	-	Monoterpenes + sesquiterpenes, resin contains diterpenes (boswellic acids)
Geranium	√	-	minor	-	Monoterpenoid alcohols (citronellol, geraniol), monoterpenoid esters
Grapefruit	√	-	minor	-	Monoterpenes (limonene), sesquiterpenoid ketones (nootkatone), monoterpenoid aldehydes
Juniper berry	√	-	√	-	Monoterpenes (*α*-pinene), sesquiterpenes (*β*-caryophyllene)
Lavender	√	-	-	-	Monoterpenoid esters (linalyl acetate), monoterpenoid alcohols (linalool)
Lemon	√	-	-	-	Monoterpenes (limonene), monoterpenoid aldehydes (citral, small %)
Lemon eucalyptus	√	-	-	-	Monoterpenoid aldehydes (citronellal), monoterpenoid alcohols (citronellol)
Lemongrass	√	-	minor	-	Monoterpenoid aldehydes (citral), monoterpenoid alcohols (geraniol)
Orange	√	-	-	-	Monoterpenes (limonene)
Palmarosa	√	-	-	-	Monoterpenoid alcohols (geraniol), monoterpenoid esters
Patchouli	-	-	√	-	Sesquiterpenoid alcohols (patchoulol)
Peppermint	√	-	-	-	Monoterpenoid alcohols (menthol), monoterpenoid ketones (menthone), monoterpenoid esters
Rosemary	√	-	minor	-	Monoterpenoid oxides (1,8-cineole), monoterpenoid ketones (camphor), monoterpenes (*α*-pinene)
Sage	√	-	-	-	Monoterpenoid ketones (thujone, camphor)
Sweet fennel	-	-	-	√	Phenylpropanoid ethers (anethole)
Tea tree	√	-	minor	-	Monoterpenoid alcohols (terpinen-4-ol), monoterpenes, minor sesquiterpenes
Wintergreen	-	-	-	√	Phenylpropanoid esters/phenolic acid esters (methyl salicylate)
Ylang ylang	√	-	√	√	Monoterpenonid alcohols (linalool), sesquiterpenes (*β*-caryophyllene), esters (benzyl acetate)

Compounds listed in parentheses represent the main chemical constituent for each essential oil. Note: resources used include [27,28].

**Table 3:** Categorization of 14 essential oils exhibiting the strongest repellency organized into five main odor groups.

Odor Category	Essential Oils
Camphor-like	Basil, Rosemary, Tea tree, Sage, Lemon eucalyptus
Minty	Peppermint, Wintergreen
Floral (sweet)	Lavender, Bergamot
Citrus	Lemon, Lemongrass, Citronella
Spicy (woody)	Cinnamon
Pine-like	Juniper berry

Note: resource used [28,29].

## Data Availability

The original contributions presented in this study are included in the article. Further inquiries can be directed to the corresponding author.

## Abbreviations

The following abbreviations are used in this manuscript:

IR3535	Ethyl butylacetylaminopropionate)
DEET	N,N-Diethyl-meta-toluamide
RT	Repellency threshold
RD_50_ value	Concentration at 50% of the population of insects in the test group were repelled
GABA	Gamma-aminobutyric acid
